# Treatment with L-citrulline in patients with post-polio syndrome: study protocol for a single-center, randomised, placebo-controlled, double-blind trial

**DOI:** 10.1186/s13063-017-1829-3

**Published:** 2017-03-09

**Authors:** Simone Schmidt, Vanya Gocheva, Thomas Zumbrunn, Daniela Rubino-Nacht, Ulrike Bonati, Dirk Fischer, Patricia Hafner

**Affiliations:** 1 Division of Neuropediatrics, University of Basel Children’s Hospital, Spitalstrasse 33, Postfach 4031 Basel, Switzerland; 2 Division of Neurology, University Hospital Basel, Basel, Switzerland; 3 Department of Clinical Research, Clinical Trial Unit, University Hospital Basel, Basel, Switzerland; 4 Division of Internal Medicine, University Hospital Basel, Basel, Switzerland; 5 Division of Neurology, Medical University Clinic, Kantonsspital Baselland, Bruderholz, Switzerland

**Keywords:** L-citrulline, Post-polio syndrome, Clinical trial, Quantitative MRI

## Abstract

**Background:**

Post-polio syndrome (PPS) is a condition that affects polio survivors years after recovery from an initial acute infection by the Poliomyelitis virus. Most often, patients who suffered from polio start to experience gradual new weakening in muscles, a gradual decrease in the size of muscles (muscle atrophy) and fatigue years after the acute illness. L-citrulline is known to change muscular metabolism synthesis by raising nitric oxide (NO) levels and increasing protein synthesis. This investigator-initiated, randomised, placebo-controlled, double-blind, trial aims to demonstrate that L-citrulline positively influences muscle function and increases muscular energy production in patients with PPS.

**Methods/design:**

Thirty ambulant PPS patients will be recruited in Switzerland. Patients will be randomly allocated to one of the two arms of the study (placebo:verum 1:1). After a 24-week run-in phase to observe natural disease history and progression, participants will be treated either with L-citrulline or placebo for 24 weeks. The primary endpoint is change in the 6-min Walking Distance Test. Secondary endpoints will include motor function measure, quantitative muscle force, quantitative muscle magnetic resonance imaging and magnetic resonance spectroscopy and serum biomarker laboratory analysis

**Discussion:**

The aim of this phase IIa trial is to determine if treatment with L-citrulline shows a positive effect on clinical function and paraclinical biomarkers in PPS. If treatment with L-citrulline shows positive effects, this might represent a cost-efficient symptomatic therapy for PPS patients.

**Trial registration:**

ClinicalTrial.gov, ID: NCT02801071. Registered on 6 June 2016.

**Electronic supplementary material:**

The online version of this article (doi:10.1186/s13063-017-1829-3) contains supplementary material, which is available to authorized users.

## Background

### Poliomyelitis and post-polio syndrome (PPS)

Poliomyelitis is an infectious viral disease that can strike at any age and affects the nervous system, resulting in paralysis with muscle spasms and, in some cases, acute encephalitis [1]. To date, most regions in the world are certified polio free. However, poliomyelitis is still endemic in two countries worldwide (Afghanistan and Pakistan) with 74 reported cases in 2015 [2].

PPS is a condition that affects polio survivors years after recovery from an initial acute infection by the Poliomyelitis virus. Most often, patients affected by polio start to experience gradual new weakening in muscles which were previously infected by the original polio infection [1].

The most common symptoms include slowly progressive muscle weakness, fatigue (both generalised and muscular) and a gradual decrease in muscle size (muscle atrophy). The diagnosis of PPS relies nearly entirely on clinical information. Physicians diagnose PPS after completing a comprehensive medical history and physical examination, and by excluding other disorders that could explain the symptoms [3].

The exact incidence and prevalence of PPS is unknown. Due to the large number of polio survivors, in 2004 250,000 patients with PPS were estimated in Europe, 20 million worldwide [4]. Ragonese et al. showed in 2005 a prevalence rate for PPS of 31% (women more than men) in patients affected by poliomyelitis [5].

The aetiology and pathogenesis of PPS are unclear. It has been hypothesised that the new weakness of PPS appears to be related to the degeneration of individual nerve terminals in the motor units. The usage of these recovered but overly extended motor units for years overstresses the neurons which, over time, lose their ability to maintain the increased work demands. This results in slow deterioration of the neurons, leading to reduced muscle strength [6, 7].

This hypothesis explains why PPS occurs after a delay and has a slow and progressive course. There are currently no effective pharmaceutical or rehabilitative treatments that can stop the deterioration or reverse the deficits caused by the syndrome itself [8].

### L-citrulline

L-citrulline (CIT) is a nonessential amino acid, not involved in protein synthesis. However, CIT plays a central part in cellular metabolism. CIT is produced but not metabolised in the gastrointestinal tract. More than 80% of the citrulline produced by the gastrointestinal tract appears to be taken up by the kidneys [9, 10]. CIT is then converted to L-arginine in the renal proximal tubules (via the enzymes arginosuccinate synthase and arginosuccinate lyase) [9]. As a precursor of L-arginine, CIT is transformed into nitric oxide (NO) by nitric oxide synthase (NOS). El-Hattab et al. demonstrated that the intake of CIT in MELAS (mitochondrial encephalomyopathy, lactic acidosis, and stroke-like episodes) patients showed a substantially higher increase of L-arginine and NO plasma concentrations than did the same dose of L-arginine [11]. Both CIT and L-arginine support NO synthesis in a variety of tissues, including muscle tissue. Osowska et al. reported that with a CIT-enriched diet, protein synthesis improved and, therefore, the protein mass in the muscle could be increased [12]. NO triggers mitochondrial biogenesis and enhances coupled respiration and adenosintriphosphate (ATP) production. This process is mediated via a cGMP (guanosine 3’,5’-monophosphate)-dependent signalling pathway that activates the expression of peroxisome proliferator-activated receptor-γ coactivator 1α (PGC-1α), which is a master regulator of mitochondrial biosynthesis [13].

Therefore, CIT supplementation results in reduced fatigue and improved endurance for both aerobic and anaerobic prolonged exercise. It seems to interact with ATP production (in a beneficial manner) by increasing the efficiency of energy production [14].

Single oral doses of CIT of up to 15 g are tolerated without any side effects in contrast to equivalent oral doses of L-arginine, where osmotic diarrhoea has been observed [15]. The same observations may be reported in our own studies (data not yet published) in which CIT (doses of up to 15 g/d) was administered to patients with Becker muscular dystrophy and Duchenne muscular dystrophy.

Unpublished results showed beneficial effects of CIT on muscle function and fatigue in muscular dystrophies. In both pathways, the stimulation of skeletal muscle protein syntheses via ATP production and mitochondrial biosynthesis stimulation could be potentially useful in other chronic neuromuscular disorders of neurogenic origin such as PPS.

## Methods/design

### Study design

The study is an investigator-initiated, randomised, placebo-controlled, double-blind, efficacy phase IIa trial and is conducted over a time period of 48 weeks. We plan to enrol 30 ambulant PPS patients aged older than 18 years. Recommendations for Interventional Trials (SPIRIT) Checklist on which the study protocol is based is presented as Additional file 1. A (SPIRIT) figure, showing the planned visit and examination schedule is presented in Fig. 2.﻿

### Inclusion criteria

In order to participate, patients are required to meet the following criteria:

be ambulant patients aged 18 years and above at the time of screening and able to walk 150 m in the 6-min Walking Distance Test (6MWD); women of child-bearing potential must be willing to use a contraceptive during the study; there must be prior paralytic poliomyelitis with evidence of motor neuron loss. This is confirmed by history of the acute paralytic illness, signs of residual muscle weakness and atrophy on neuromuscular examination, and signs of motor neuron loss.

A period of partial or complete functional recovery after acute poliomyelitis, followed by an interval (usually 15 years or more) of stable neuromuscular function is required and a slowly progressive and persistent new muscle weakness or decreased endurance, with or without generalised fatigue, muscle atrophy or muscle and joint pain must be demonstrable. Symptoms must have persisted for at least 1 year. Patients with other neuromuscular, medical and skeletal abnormalities causing the symptoms are excluded.

### Exclusion criteria

Excluded are patients those who have previously (3 months or less) or concomitantly participated in any other therapeutic trial; the use of CIT or L-arginine within the last 3 months; a known individual hypersensitivity to CIT; a known or suspected malignancy; any other chronic disease or clinically relevant limitation of renal, hepatic or cardiac function at the discretion of the investigator; pregnant or breast-feeding women and patients with severe renal failure (calculated glomerular filtration rate <30 ml/min).

### Randomisation and blinding

Patients who meet the study admission criteria are enrolled in the study and a single subject identification number will be assigned. Patients are allocated to the two study groups, CIT or placebo, in a 1:1 ratio. The trial is double blind, with both patients as well as the investigators assessing outcomes blinded to treatment allocation. Dropouts after visit 3 (week 24) will not be replaced.

### Intervention

**Fig. 1:**
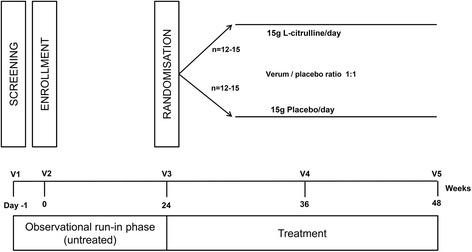
Flow chart showing the study design

All patients will undergo a 6-month observational (untreated) period to observe the natural history of their condition. Afterwards, they will be randomised into a verum or a placebo (1:1) group. All patients randomised to the active compound receive a daily dose of 15 g CIT (Hänseler AG, Herisau, Switzerland) separated in three doses per day (3 × 5 g). The patients randomised to the placebo group will receive matching placebo (sachets look like verum but contain mannitol). Treatment is given for a period of 24 weeks. At baseline (visit 2), week 24 (visit 3) as well as at the end of the study (visit 5), clinical measures, laboratory and magnetic resonance imaging (MRI) measures will be performed. This includes the 6MWD, the Motor Function Measurement (MFM) scale, quantitative motor tests (QMT), motor function assessment using quantitative thigh muscle MRI, and magnetic resonance (MR) spectroscopy and laboratory blood analysis. Furthermore, assessments concerning quality of life and impairments concerning daily activities are done with standardised questionnaires (SIPP – Self-reported Impairments in Persons with late effects of Polio, IBM-FRS – Inclusion Body Myositis Functional Rating Scale; WHOQOL – The WHO Quality of Life – BREF Questionnaire). A flow chart showing the study design is provided in Fig. 1.

To compare results of quantitative muscle MRI (Iterative Decomposition of water and fat with Echo Asymmetry and Least-squares estimation-Carr-Purcell-Meiboom-Gill (IDEAL-CPMG) with T_2_ and lipid quantitation and dynamic ^31^P-MR spectroscopy), motor function and serum markers of muscle necrosis, oxidative and nitrosative stress with an age- and gender-correlated group of people, up to 20 subjects not suffering from neuromuscular disorders will be included into a healthy control group. In this population, the 6MWD, MFM scale, QMT, quantitative MRI and MR spectroscopy and a blood test will be performed at a single time point.

### Study procedure

At screening (visit 1, day −1), patients are informed about preclinical data, alternative treatments, risks and possible benefits of the study. Further, written informed consent is obtained. After signing the Informed Consent Form, inclusion and exclusion criteria are verified. If the criteria are fulfilled, the patient will be enrolled in the study. During the screening visit, the following procedures are performed: 6MWD; vital signs; physical examination; MFM scale. After screening, four visits will be scheduled at baseline (week 0, visit 2), week 24 (visit 3), week 36 (visit 4) and week 48 (visit 5, end of study).

**Fig. 2:**
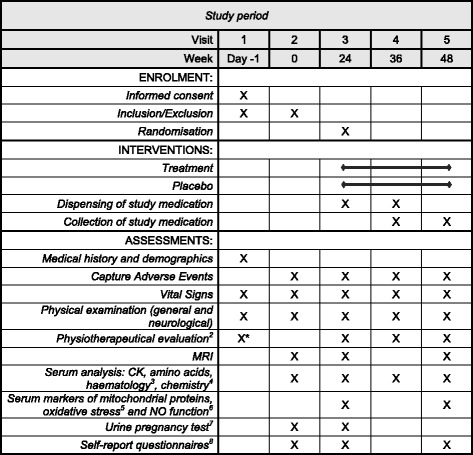
Planned visiting and examination schedule *will be used as baseline values. Vital signs include: blood pressure, heart rate, weight at every visit; height only at screening visit. Motor Function Measurement (MFM) scale, 6-min Walking Distance Test (6MWD), quantitative motor tests (QMT), and screening will be used as baseline values. Full blood count: erythrocytes, reticulocytes, leucocytes, platelets, haemoglobin, haematocrit. GOT, GPT, creatinine, electrolytes (Na^+^, K^+^, Ca^2+^), urea, creatine kinase (CK), glycosylated haemoglobin (HbA1c), cholesterol, high-density lipoprotein (HDL), low-density lipoprotein (LDL), triglycerides, calculated glomerular filtration rate (GFR). Oxidised DNA (8-hydroxy-2’-deoxyguanosine: 8OHDG), carbonylated proteins (4-Hydroxynonenal (4-HNE)), mitochondrial proteins (citratsynthase, cytochrome C oxidase subunit 1, succinate dehydrogenase subunit A), nitrotyrosine, cGMP. Woman of child-bearing potential. SIPP – Self-reported Impairments in Persons with late effects of Polio, IBM-FRS – Inclusion Body Myositis Functional Rating Scale, WHOQOL-BREF – The WHO Quality of Life – BREF Questionnaire

During visit 2 (week 0, baseline), the following procedures are performed: check inclusion/exclusion criteria; vital signs; physical examination; self-report questionnaires; muscle MRI; adverse events; blood draw (laboratory analysis) and urine pregnancy test in women of child-bearing potential. If the patient still qualifies for the study, they will be definitively enrolled in the study. Two weeks after screening, self-report questionnaires are filled in a second time by patients to evaluate the questionnaires for test-retest reliability. During visit 3 (week 24), the following procedures will be performed: 6MWD; vital signs; physical examination; self-report questionnaires; MFM scale; QMT; muscle MRI; adverse events; blood draw (laboratory analysis) and urine pregnancy test in women of child-bearing potential. If the patient still qualifies for the study, they will be randomised and receive the study medication or placebo accordingly. During visit 4 (week 36), the following procedures are performed: 6MWD; vital signs; physical examination; MFM scale; QMT adverse events; blood draw (laboratory analysis); dispensing of study medication; collection of boxes with sachets of used study medication for compliance control. During the last visit (visit 5, week 48, end of study), the following procedures are performed: 6MWD; vital signs; physical examination; self-report questionnaires; MFM scale; QMT; muscle MRI; adverse events; blood draw (laboratory testing); collection of boxes with sachets of used study medication for compliance control. The full planned visiting and examination schedule is provided in Fig. 2.

### Quality assurance

To assess high-quality conduct of the trial in accordance with the protocol, all medical staff involved in this study are certified in Good Clinical Practice (GCP). The physiotherapists who perform the tests were trained and certified in Lyon where the MFM scale has been established and validated. To assess compliance of medication intake, empty and full boxes with sachets are returned by the patients at visits 4 and 5.

Allocation concealment is assured by an unblinded pharmacist who has access to the randomisation list. The investigator and the participants stay blinded until the end of the study.

### Safety assessments

Adverse events are monitored throughout the study. At every study visit, patients are asked about adverse events. At visits 1, 3, 4 and 5, a physical examination will be performed and the vital parameters will be measured. The following safety parameters are checked: full blood count; blood chemistry (transaminases, creatinine, electrolytes, urea); marker of muscle necrosis (creatine kinase concentration); serum markers of oxidative stress (8-hydroxy-2’-deoxyguanosine (8OHDG), carbonylated proteins); serum markers of nitrosative stress (nitrotyrosine, cGMP); all amino acids; glucose metabolism marker (HbA1c); lipid metabolism markers (cholesterol, high-density lipoprotein (HDL), low-density lipoprotein (LDL), triglycerides). If pathological changes independent of the known muscle disease should be detected, the affected patients will be informed immediately and the possibilities of further investigation or treatment of these abnormalities according to current medical knowledge, respectively, will be discussed.

Withdrawal of consent, protocol violations caused by the patients (noncompliance), logistical reasons, inability to attend study visits as defined in the protocol, abnormal laboratory results and abnormal increase in blood pressure as determined by the investigator can lead to an early termination of the study. Patients with adverse reactions which have occurred in context of the study are followed up by the investigator up to 30 days after the last visit. Serious adverse events will be reported within 7 days (after being reported by the patient) to the local national Ethics Committee. Female patients of child-bearing age will be instructed to use a double-barrier method of birth control and to immediately inform the investigator if they become pregnant during the study. The study drug is discontinued at the discretion of the investigator.

### Outcome measures

#### Primary outcome measure

##### Change of 6MWD from week 24 to week 48 under L-citrulline therapy versus placebo

Several tests have been reported in the medical literature to assess muscle strength and functional ability, to monitor the progression of the disease, and to evaluate the results of drug interventions and rehabilitation. Timed clinical functional assessments include the 6-min Walking Distance Test (6MWD) in meters which is helpful to assess muscle function and fatigue in patients with neuromuscular disorders [16, 17]. The 6MWD is a validated tool to measure the distance that an individual is able to walk over a total of 6 min on a hard, flat surface. The goal for the individual is to walk as far as possible in 6 min. The individual is allowed to self-pace and rest as needed as they traverse back and forth along a marked walkway.

#### Secondary outcome measures

##### Change of MFM scale total score

The MFM scale, a validated assessment tool to measure motor function in both ambulant and nonambulant patients with neuromuscular disorders, was developed in France in 2005. It includes 32 items that evaluate three dimensions of motor performance, including specific motor functions, such as transfers and standing posture (D1), proximal and axial (D2) and distal (D3) and a total MFM score involving all of the motor dimensions. The items are scored and summed to comprise a total score where the maximum represents normal motor function. The instruction manual, validation examinations and other publications using the MFM scale can be downloaded at the MFM scale website [18].

##### Change of quantitative muscle MRI (IDEAL-CPMG with T_2_ and lipid quantitation and dynamic ^31^P-MR spectroscopy)

One of the advantages of muscle MRI imaging methods is that the raw data can be stored and direct comparisons from data at several time points are possible. Visual and semiquantitative muscle MRI studies have been applied in inherited neuromuscular disorders, including SMA, and have identified anatomical abnormality, such as absent, aberrant, or accessory muscles, changes in tissue composition, such as fatty infiltration or increased water content, and masses or mass-like lesions [19, 20]. We use the IDEAL-CPMG sequence allowing simultaneous T_2_ and lipid quantitation and dynamic Phospho-magnet resonance (^31^P-MR) spectroscopy that quantifies the kinetics of phosphocreatine (PCr), ATP and intracellular pH in lower leg muscles during rest, exercise and recovery. As this sequence is not tested previously in an age-correlated group of people without neuromuscular disorders, up to 20 age- and gender-correlated subjects will be examined additionally at one time point.

In neuromuscular disorders, quantitative muscle MRI (qMRI) detects disease progression more sensitively compared to other clinical scores. Excellent reproducibility of qMRI was demonstrated in healthy volunteers as well as in patients [21, 22]. qMRI was more sensitive than clinical evaluation and visual analysis of MRI scans to detect disease progression [20, 23, 24].

##### Change of serum concentrations for markers of muscle necrosis (creatine kinase), oxidative stress (8OHDG, carbonylated proteins 4-Hydroxynonenal (4-HNE)), nitrosative stress (nitrotyrosine, cGMP), and mitochondrial-related genes (*Citratsynthase*, *Cytochrome C oxidase subunit 1*, *Succinate dehydrogenase subunit A*)

Our treatment aims at a stimulation of NO concentrations measured by indirect markers (nitrotyrosine, cGMP), an improvement of the muscular energy situation (citratsynthase, cytochrome C oxidase subunit 1, succinate dehydrogenase subunit A) with reduction of oxidative stress (8OHDG in urine, carbonylated proteins) and a slowing of muscle degeneration (levels of creatine kinase, transaminases, alkaline phosphatase).

#### Auxiliary outcome measures

##### Change of quantitative muscle test (QMT) of elbow flexion, hand grip, knee extension and ankle extension using hand-held dynamometry (HHD)

As the distribution of muscle weakness in PPS patients varies widely, a quantitative muscle testing, including different muscles of the upper and lower limbs, provides probably a more detailed insight into the distribution and evaluation of muscle weakness in each patient.

##### Change of self-report questionnaires SIPP, IBM-FRS and WHOQOL – BREF

The Self-reported Impairments in Persons with late effects of Polio (SIPP) is a new rating scale first published in 2013 and recently reevaluated for test-retest reliability [25, 26]. The IBM-FRS (Inclusion Body Myositis Functional Rating Scale) and the WHOQOL (World Health Organisation – Quality of Life) – BREF questionnaire are widely used and validated scales to assess motor function especially in patients with asymmetric neuromuscular conditions, such as inclusion body myositis, and/or quality of life regardless of any underlying condition [27]. Our aim is to revalidate test-retest reliability of these tests in our patient group and to assess changes compared to other clinical and subclinical outcome measures.

### Randomisation scheme

The study aims to include 15 patients in each of two treatment arms (placebo and verum). A randomisation scheme called Big Stick Design [28] with a maximum tolerated imbalance (MTI) between groups of two patients will be applied. The randomisation corresponds to tossing a fair coin as long as the difference in group sizes does not exceed the MTI. If the MTI is reached, a deterministic allocation is performed so that the difference in group sizes is reduced. For emergency breaking of the group assignment, unblinding envelopes are provided. These envelopes have a window in which only the running patient’s ID is visible and contain an unblinding form that states the patient’s group assignment.

### Sample size estimation

It is assumed that patients in the verum group will on average have a 25% longer 6MWD after 24 weeks of treatment with CIT compared to baseline. It is further assumed that patients in the placebo group will on average have no improvement, but also no deterioration with regard to the 6MWD over the 24 weeks of placebo treatment. For sample size estimation, a data set from a study of patients with Becker’s muscular dystrophy (BMD) treated with the same CIT dose for 6 weeks was used (performed at the University of Basel Children’s hospital, publication in preparation). Patients (mean age 31 years) were assigned to CIT treatment for the duration of 6 weeks and their 6 MWD was measured at baseline and follow-up. Using a resampling method, each sample size *n*_i=1,…,41_ = 10, …, 50 was evaluated by simulating 999 times *n*_i_ patients with their 6MWD baseline and follow-up values from a multivariate normal distribution. The multivariate normal distribution was parameterised by the mean 6MWD at baseline from the BMD data set, the mean 6MWD at follow-up (mean baseline value augmented by the expected relative increase *θ*_i=1,…41_ = 0.1, …, 0.5), and the variance-covariance matrix as estimated from the BMD data set. The patients were randomised in a 1:1 or 1:2 ratio to the placebo and the verum group, respectively, in order to examine the effect of the randomisation ratio on sample size. The resulting data set was analysed with an analysis of covariance (ANCOVA) with 6MWD follow-up measurement as response, 6MWD baseline measurement as adjusting covariate and treatment group (placebo versus verum) as explanatory variable of interest. Finally, it was tested whether there was a difference between the placebo and verum groups.

**Fig. 3:**
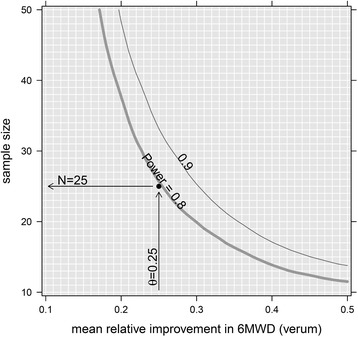
Randomisation placebo:verum 1:1. Association of sample size (*N*) and the mean relative improvement of the verum group in the 6-min Walking Distance Test at follow-up (*θ*), assuming no improvement in the placebo group, for a randomisation ratio of 1:1 into the placebo and verum groups, respectively. Curves are smoothed for illustration purposes only

For an expected mean increase in 6MWD of 25% in the verum group, 28 subjects need to be recruited to obtain 25 evaluable patients, assuming a dropout rate of 10% and when randomising patients 1:1 to placebo and verum, respectively (Fig. 3). To further increase the sensitivity, a similar 24-week run in observational period will be performed in all patients before active or placebo treatment. The latter should help within the placebo group to separate natural history changes from placebo effects during the treatment period.

### Statistics

The full analysis set (FAS) consists of all patients who are randomised and for whom a baseline measurement of the primary endpoint is available. The intention-to-treat (ITT) set is a subset of the FAS that consists of all patients for whom there is a follow-up measurement of the primary endpoint. According to the ITT principle, each patient will be analysed according to the treatment that they are randomly allocated to. The per-protocol (PP) set is a subset of the ITT set that consists of all patients with complete follow-up measurements of the primary endpoint. Each patient will be analysed according to the treatment that they actually receive.

An ANCOVA will be used for the primary analysis. The response variable is the 6MWD after the 24-week treatment period (follow-up). The explanatory variables are the 6MWD at the onset of the treatment period (baseline) and the group (placebo versus verum). The model estimates will be presented together with 95% confidence intervals and *p* values with respect to the null hypothesis that the corresponding estimate has a value of 0. The analysis will be performed on the ITT set. The analysis will be repeated on the PP set and compared to the ITT analysis as a sensitivity analysis.

Following the analysis of the primary endpoint, the secondary endpoints will be analysed as outlined above for the primary endpoint.

### Quality control

Monitoring of the study will be performed by an independent person (KMS Monitoring, Zug). All inclusion and exclusion criteria will be checked, whether the data have been recorded correctly in the CRF, whether the drug accountability is correct and whether any serious adverse events (SAEs) have occurred during the study.

### Ethical considerations

PPS is a serious progressive neuromuscular disease with no therapy so far. Affected patients suffer from severe impairment that justifies an attempt to improve this condition using an almost side-effect-free treatment.

The participation in this study is voluntary. If the patients do not want to participate, they will not experience any disadvantages concerning any further medical treatment. The same applies if the patients withdraw the consent at a later time point. They have this possibility at any time. Possible withdrawal of consent from the study can occur without giving any reason. In case of withdrawal the data collected until this time point will be used and the samples (blood) collected in the context of the study will be destroyed. In case of withdrawal the patient will undergo a final visit for a medical examination for his own safety.

A placebo group is necessary to demonstrate the efficacy of the used medication (CIT) and to exclude a possible placebo effect.

There is a great potential that positive results of this study could lead to other large multicenter trials. Furthermore, if we can show an effect of CIT on muscle function, this will be a major breakthrough and mean a better symptomatic treatment of the muscle degeneration that occurs in PPS.

## Discussion

The aim of this investigator-initiated, randomised, placebo-controlled, double-blind, phase IIa trial is to examine whether treatment with CIT has a beneficial effect on clinical muscle function and fatigue and on the surrogate biomarkers that assess muscle ATP production and mitochondrial function in PPS. If this turns out to be the case, a cost-effective, symptomatic therapy with CIT could be available for these patients.

As PPS is a very heterogeneous disease, all patients will undergo a 24-week observational (untreated) period to observe the natural history of the disease. For the primary endpoint the 6MWD was chosen, which implies that all included patients are still ambulatory. As physical fatigue is one of the major and most limiting symptoms [3], the 6MWD seems to be the ideal endpoint to observe positive effects on muscle function, and to assess fatigue and endurance due to CIT intake. Furthermore, due to the asymmetric nature of the disorder the 6MWD seems to be optimal regardless of whether walking impairment is caused by the right or left proximal or distal leg being affected.

The MFM is a scale used in neuromuscular diseases to assess their severity and progression. It is used by physiotherapists or rehabilitation physicians and is useful to observe motor function changes in clinical trials [29]. The test includes 32 items including specific motor functions such as transfers and standing (D1), proximal and axial (D2) and distal (D3). The total MFM score involves all the motor dimensions [18] and can, therefore, also detect motor function changes in body parts which are not involved in walking and are not evaluated in the 6MWD.

In order to increase sensitivity and to detect subclinical positive effects on muscle mass and muscle metabolism, quantitative muscle MRI (qMRI) is performed. We and others have demonstrated that qMRI is more sensitive than clinical evaluation and visual analysis of MRI scans and it also shows an excellent reproducibility [20, 21, 24]. qMRI is a powerful technique to evaluate neuromuscular and muscular disorders in a noninvasive way for evaluation of disease progression and, furthermore, it is a good biomarker in clinical trials [30]. Kinali et al. showed a good correlation between muscle histology and MRI changes in patients with DMD [31]. Furthermore, to investigate ATP in human muscle in vivo, ^31^P-MR spectroscopy is used (during rest, exercise and recovery). With this method, the mitochondrial function and the regeneration of ATP after muscle exercise could be investigated.

### Trial status

The trial started enrolment in June 2016 and is expected to be completed by the end of August 2017.

## Additional file

Additional file 1: Recommended items to address in a clinical trial protocol and related documents. (PDF 57 kb)

## Abbreviations

^31^P-MR: Phospho-magnet resonance
4-HNE: 4-Hydroxynonenal
6MWD: 6-min Walking Distance Test
8OHDG: 8-hydroxy-2’-deoxyguanosine
ATP: Adenosintriphosphate
BMD: Becker muscle dystrophy
BSD: Big Stick Design
cGMP: Guanosine 3’,5’-monophosphate
CIT: L-citrulline
GFR: Glomerular filtration rate
HDL: High-density lipoprotein
IBM-FRS: Inclusion Body Myositis Functional Rating Scale
IDEAL-CPMG: Iterative Decomposition of water and fat with Echo Asymmetry and Least-squares estimation-Carr-Purcell-Meiboom-Gill
LDL: Low-density lipoprotein
MELAS: Mitochondrial encephalomyopathy, lactic acidosis and stroke-like episodes
MFC: Muscle fat content
MFM: Motor Function Measurement
MRI: Magnetic resonance imaging
MTI: Maximum tolerated imbalance
NO: Nitric oxide
NOS: Nitric oxide synthase
PCr: Phosphocreatine
PGC-1α: Peroxisome proliferator-activated receptor-γ coactivator 1α
PPS: Post-polio syndrome
qMRI: Quantitative magnetic resonance imaging
SIPP: Self-reported Impairments in Persons with late effects of Polio rating scale
WHOQOL: The World Health Organisation - Quality Of Life
